# Influence of age and sex on the diagnostic accuracy of pleural fluid carcinoembryonic antigen for malignant pleural effusion: a *post hoc* analysis

**DOI:** 10.3389/fonc.2025.1549621

**Published:** 2025-05-06

**Authors:** Dan-Ni Yang, Jian-Xun Wen, Yan Niu, Ling Hai, Qian Yang, Su-Na Cha, Cheng Yan, Hong-Zhe Zhu, Ting-Wang Jiang, Li Yan, Jin-Hong Huang, Hong Chen, Xin-Ru Zuo, Wen-Qi Zheng, Zhi-De Hu

**Affiliations:** ^1^ Department of Laboratory Medicine, the Affiliated Hospital of Inner Mongolia Medical University, Hohhot, China; ^2^ Key Laboratory for Biomarkers, Inner Mongolia Medical University, Hohhot, China; ^3^ Medical Experiment Center, the College of Basic Medicine, Inner Mongolia Medical University, Hohhot, China; ^4^ Department of Pathology, the College of Basic Medical, Inner Mongolia Medical University, Hohhot, China; ^5^ Department of Pathology, the Affiliated Hospital of Inner Mongolia Medical University, Hohhot, China; ^6^ Department of Key Laboratory, the Changshu Hospital of Nantong University, Changshu, China; ^7^ Department of Respiratory and Critical Care Medicine, the Affiliated Hospital of Inner Mongolia Medical University, Hohhot, China; ^8^ Department of Respiratory and Critical Care Medicine, the Changshu Hospital of Nantong University, Changshu, China; ^9^ Inner Mongolia Medical University, Hohhot, China

**Keywords:** pleural fluid, carcinoembryonic antigen (CEA), malignant pleural effusion (MPE), age, sex

## Abstract

**Introduction:**

The diagnostic value of pleural fluid carcinoembryonic antigen (CEA) for malignant pleural effusion (MPE) has been well recognized. However, whether age and sex affect its diagnostic accuracy remains unknown. This study aimed to investigate the influence of age and sex on the accuracy of CEA for differentiating MPE and benign pleural effusion (BPE).

**Methods:**

We enrolled participants from a prospective cohort (SIMPLE) and a retrospective cohort (BUFF). All participants were patients with pleural effusion with undetermined causes. The clinical characteristics were extracted from the medical records of participants. The area under receiver operating characteristics (ROC) curve (AUC) was used to estimate the diagnostic accuracy of CEA. The effects of sex and age on the diagnostic accuracy of CEA for MPE were analyzed using subgroup analysis. A method characterized by resampling participants with different upper limits was also used to investigate the effect of age.

**Results:**

The SIMPLE cohort included 210 participants (91 patients with MPE and 119 patients with BPE), and the BUFF cohort included 235 participants (50 patients with MPE and 185 patients with BPE). Sex did not significantly affect the diagnostic accuracy of CEA. Subgroup analyses with both cohorts revealed the decreased AUC in older patients. The resampling method revealed decreased AUC with the advancement of age.

**Conclusions:**

Age should be considered when interpreting the diagnostic accuracy of pleural fluid CEA for MPE, while the effects of sex can be ignored.

## Introduction

Pleural effusion is a common clinical condition that can be caused by more than 50 disorders, with the most common causes being tuberculous pleurisy, heart failure, pneumonia, and malignancy (1, 2). Malignant pleural effusion (MPE) is caused by primary or metastatic pleural tumors (3). Lung cancer, breast cancer, mesothelioma, lymphoma, and gastrointestinal tumors are common tumors causing MPE (4, 5). Pleural effusion is a hallmark of advanced tumor stage and thus serves as an independent prognostic factor in various cancers. The median survival of MPE patients is less than 1 year (6), depending on the type of primary cancer (7). An accurate and timely diagnosis is a prerequisite for the management of MPE. The gold standard for diagnosis of MPE is pleural biopsy or effusion cytology (2, 8–10). Cytology has 100% specificity, but its sensitivity is less than 60% (11). Therefore, patients with negative cytology results should undergo imaging-guided or thoracoscopic pleural biopsy. Imaging-guided pleural biopsy has high diagnostic accuracy, but the sampling error is problematic (12). Thoracoscopy provides direct visualization of the pleural cavity and thus has fewer sampling errors and high diagnostic accuracy (13, 14). However, both thoracoscopic and imaging-guided biopsies are invasive, and operation-related complications are challenging (15–17). In addition, the accuracy of cytology and pleural biopsy largely depends on the operator’s and observer’s experience.

With the advantages of low cost, easy access, rapidity, and objectivity, pleural fluid tumor markers represent an alternative diagnostic tool for MPE (18–21). Pleural fluid carcinoembryonic antigen (CEA) is a well-recognized diagnostic marker for MPE (22–24). It has a sensitivity of 54% and specificity of 94% for diagnosing MPE, as indicated by a systematic review and meta-analysis (25).

Notably, serum CEA increases with age (26, 27), and men have higher serum CEA than women (28). Our previous studies revealed that age affected the diagnostic accuracy of NT-proBNP (29), adenosine deaminase (ADA) (30), and cancer ratio (31) in patients with pleural effusion. Here, we hypothesized that the diagnostic accuracy of CEA can be affected by age and sex. This study aimed to investigate the effects of age and sex on the diagnostic accuracy of pleural fluid CEA for MPE and followed the Standards for Reporting of Diagnostic Accuracy Studies (STARD) guidelines (32).

## Methods

### Participants

Participants in this study were enrolled from the SIMPLE (Study Investigating Markers in PLeural Effusion) and the BUFF studies. The SIMPLE was a prospective and double-blind diagnostic study to investigate the accuracy of pleural biomarkers in patients with pleural effusion. The details of the study design have been described previously (33). We recruited patients with undiagnosed pleural effusion from two centers in China. The first center is the Affiliated Hospital of Inner Mongolia Medical University (September 2018 to July 2021), and the second is the Changshu Hospital of Nantong University (June 2020 to July 2021). The BUFF was a retrospective study. The details of the study have been introduced in our previous work (34). Briefly, we reviewed the medical records of patients with undiagnosed pleural effusion who visited the Affiliated Hospital of Inner Mongolia Medical University between July 2017 and July 2018. The patient’s clinical characteristics and final diagnosis were extracted from the medical records.

The inclusion criteria in the BUFF and SIMPLE studies were patients with undiagnosed pleural effusion who underwent a diagnostic thoracentesis. Pleural effusion was identified by chest x-ray, computed tomography (CT), or ultrasound. The exclusion criteria were: (i) patients with pleural effusion within the previous three months but its etiology was clear; (ii) patients who developed pleural effusion during hospitalization; (iii) age < 18 years; (iv) pregnancy; (v) pleural effusion caused by trauma or surgery. We performed this study according to the Declaration of Helsinki. All the participants in the SIMPLE study signed an informed consent form. The need for written informed consent to participate was waived by the Ethics Committees of the Affiliated Hospital of Inner Mongolia Medical University for the BUFF study due to the retrospective nature of the study.

### Diagnostic criteria

The diagnostic criteria have been described in our previous studies (33). Briefly, MPE was diagnosed by effusion cytology or pleural biopsy. In participants with negative cytology but who were unable or unwilling to receive pleural biopsy, the diagnosis of MPE is made by the presence of late-stage cancer with the exclusion of other benign pleural diseases. Tuberculous pleural effusion (TPE) was diagnosed using Ziehl–Neelsen staining, bacterial culture, nucleic acid amplification test, pleural biopsy, or response to anti-tuberculosis treatments (23). Parapneumonic pleural effusion (PPE) was defined by clinical characteristics, effusion bacterial culture, medical imaging characteristics (encapsulation), pleural biopsy, and response to antibiotics therapy (24). Heart failure was diagnosed by clinical characteristics, biochemistries (serum NT-proBNP and Light’s criteria), and treatment response to diuretics. Two senior clinicians (Zhi-De Hu and Li Yan) made the final diagnosis for each participant by reviewing their medical records. Any disagreements were resolved by consensus.

### Data extraction

We extracted data on the demographic characteristics, pleural fluid, and serum biochemistries on admission from the patient’s medical record. Only the first results were used for analysis if a patient underwent multiple serum and pleural fluid chemistry tests. Pleural fluid CEA in both cohorts was measured by the Architect I2000SR immunoassay analyzer (Abbott Laboratories, USA). The laboratory technicians who performed the CEA tests were blinded to the final diagnosis of the participants. This study was approved by the Ethics Committees of the Affiliated Hospital of Inner Mongolia Medical University (NO. 2018011; 2021014) and the Changshu Hospital of Nantong University (2020-KY-009).

### Statistical analysis

Continuous variables were expressed as median and interquartile range (IQR). Categorical variables were expressed as absolute numbers and percentages. We used the Shapiro–Wilk method to test the normality of continuous variables. The Mann–Whitney U test or independent Student’s t-test was used to compare continuous variables. The Chi-square test was used to compare categorical variables. We used a receiver operating characteristics (ROC) curve to measure the diagnostic accuracy of CEA, and the area under the curve (AUC) was used to measure the diagnostic accuracy of CEA. We used two different methods to investigate the effects of age on the diagnostic accuracy of CEA. First, participants were categorized into two subgroups according to their age (≤55 *vs.* >55 years old). We used DeLong’s method to compare the AUCs (35). Second, we resampled the participants with different upper limits for age and calculated the AUC of CEA in each resample dataset, as described in our previous studies (29–31). All statistical analyses and graphs were performed using R (4.3.2 version). A *p-*value <0.05 indicated statistical significance.

## Results

### Characteristics of the participants

The selection process for the participants has been described in our previous studies (34, 36). A total of 445 patients were enrolled, with 210 (91 patients with MPEs and patients with 119 BPEs) from the SIMPLE and 235 (50 patients with MPE and 185 patients with BPE) from the BUFF. The clinical characteristics of the participants are shown in **Table 1**.

**Table 1:** Characteristics of the participants.

Variables	SIMPLE cohort (n=210)			BUFF cohort (n=235)		
	MPE (n=91)	BPE (n=119)	p	MPE (n=50)	BPE (n=185)	p
Age, years	73 (67–79)	71 (61–80)	0.18	64 (54–72)	70 (58–77)	0.03
Sex, male%	55 (60)	80 (67)	0.38	26 (52)	130 (70)	0.02
Pleural fluid						
WBC, 10^6^/mL	925 (648–1,543)	858 (366–2,167)	0.72	1631 (747–2,292)	940 (407–2,176)	0.09
LDH, U/L	291 (189–468)	217 (109–459)	<0.01	357 (219–604)	232 (115–689)	0.06
Glucose, mmol/L	6.0 (5.2–6.7)	5.8 (4.7–7.0)	0.54	5.3 (3.5–6.7)	5.7 (4.1–6.8)	0.22
ADA, U/L	9 (6–13)	13 (5–36)	0.06	10 (7–21)	16 (7–36)	0.06
Protein, g/L	39 (33–44)	34 (19–45)	0.03	30 (21–42)	24 (16–38)	0.05
CEA, ng/mL	47 (4−358)	1 (1−2)	<0.01	18 (2−740)	1 (1−2)	<0.01
Serum						
Protein, g/L	64 (59−68)	60 (55−68)	0.03	63 (60−70)	62 (57−68)	0.31
LDH, U/L	217 (179−262)	206 (172−255)	0.16	203 (176−262)	213 (176−266)	0.75

Data are presented as the median (25th–75th centile) or absolute number (percentage). WBC, white blood cell; LDH, lactate dehydrogenase; ADA, adenosine deaminase; CEA, carcinoembryonic antigen.Factors affecting the diagnostic accuracy of CEA.

### Factors affecting the diagnostic accuracy of CEA

**Figure 1:**
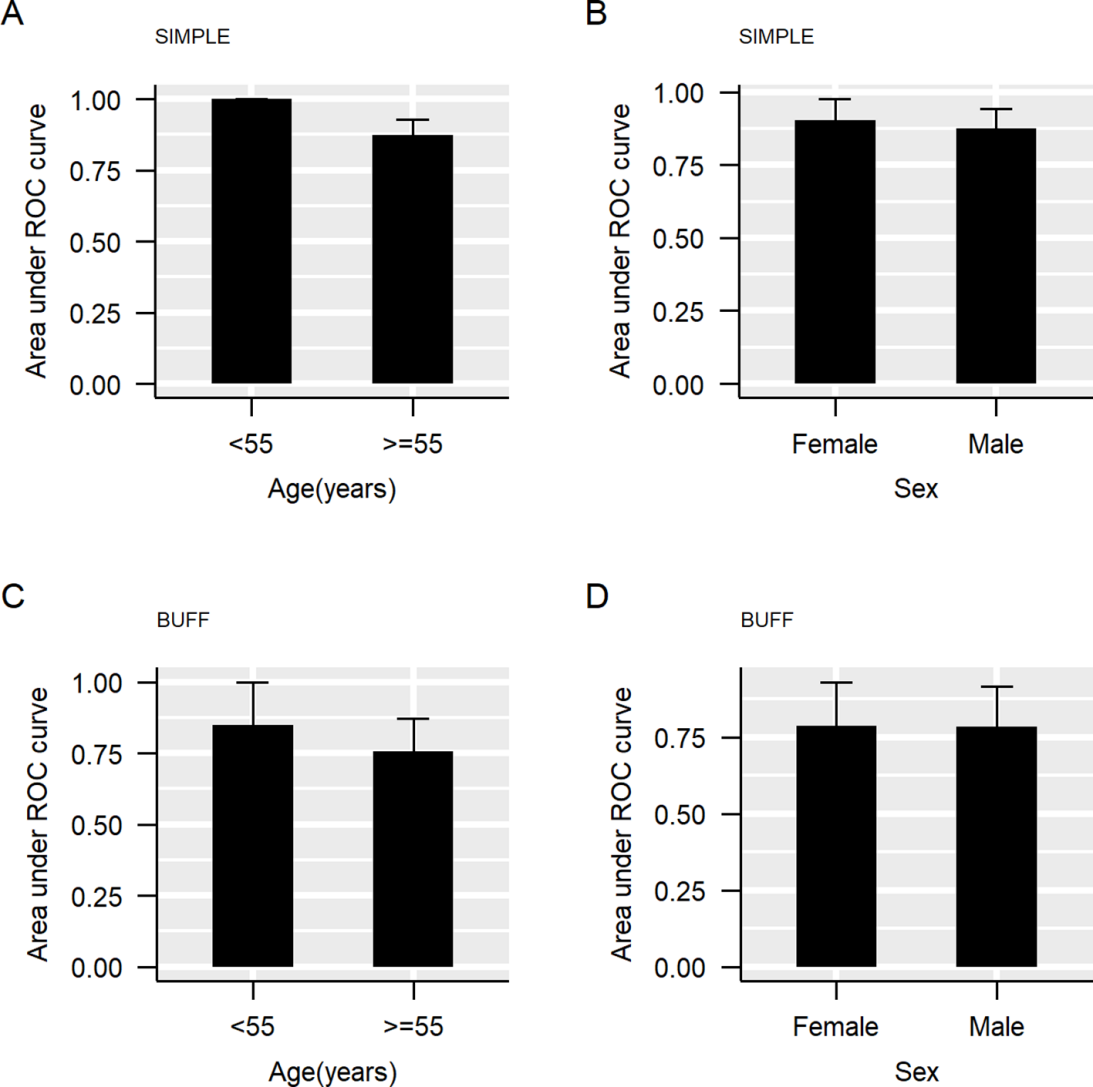
The area under the curve of carcinoembryonic antigen (CEA) in subgroups. The bar represents the 95% confidence interval (CI) of the area under the curve (AUC). **(A)**, AUC grouped by age in SIMPLE; **(B)**, AUC grouped by sex in SIMPLE; **(C)**, AUC grouped by age in BUFF; **(D)**, AUC grouped by sex in BUFF.

We divided our cohorts into two subgroups based on age, setting a threshold of 55 years, similar to our previous study (37). **Supplementary Table 1** summarizes the clinical features of the participants in each subgroup. The SIMPLE cohort was composed of 26 participants (six patients with MPE and 20 patients with BPE) aged ≤55 years and the BUFF cohort was composed of participants (14 patients with MPE and 39 patients with BPE) aged ≤55 years. **Figure 1** shows the results of subgroup analyses according to sex and age. In both the SIMPLE and the BUFF, young participants (≤55 years old) had a higher AUC than old patients (>55 years old) (**Figures 1A, C**, *p*<0.001 in the SIMPLE and *p*=0.377 in the BUFF). We did not observe any effects of sex on the AUC of CEA (**Figures 1B, D**, *p*>0.05 for both).

**Figure 2:**
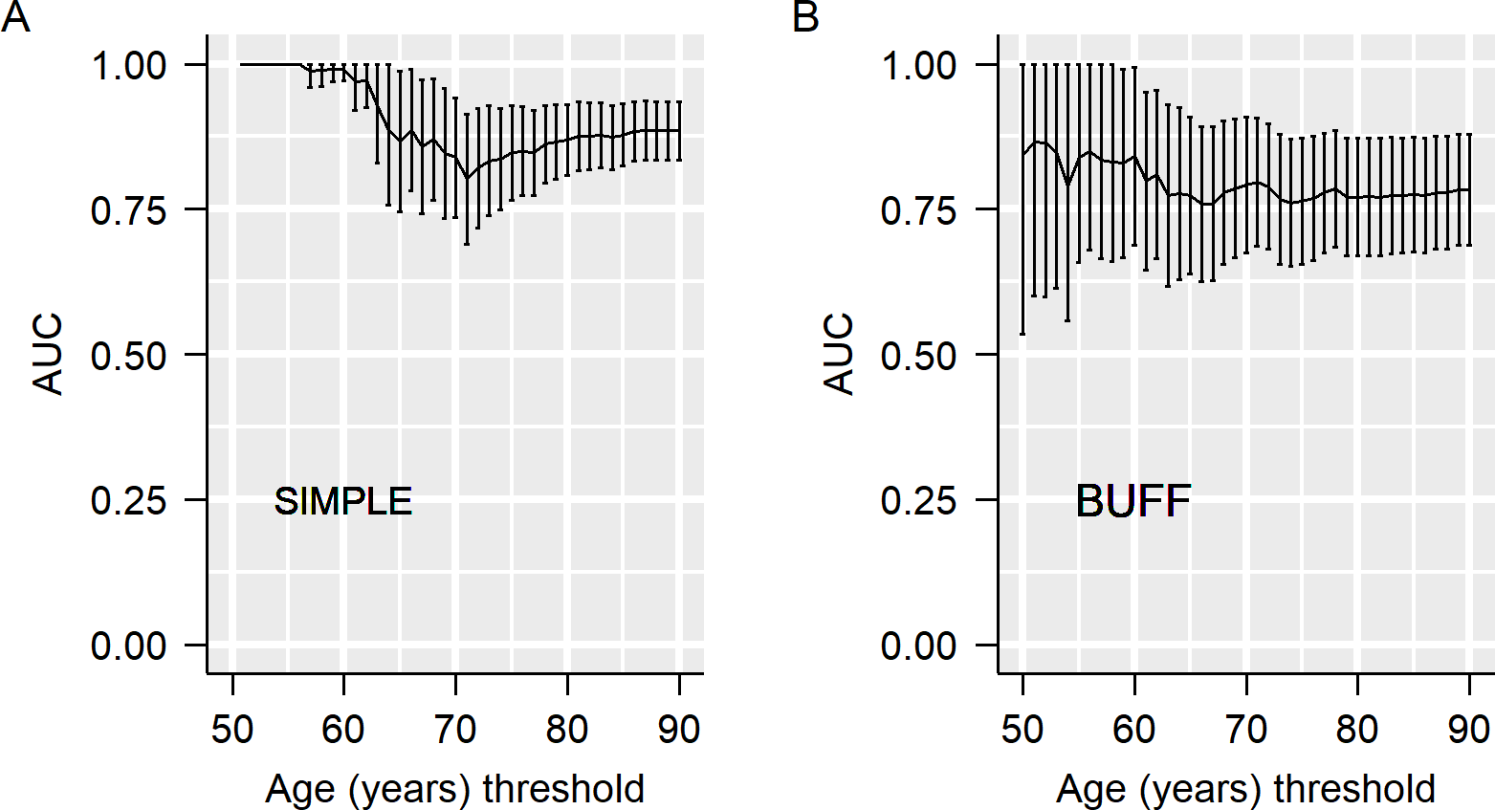
Effects of age on the area under the curve (AUC) of carcinoembryonic antigen (CEA).

Next, we used the resampling method to estimate the effects of age on the AUC of CEA. With different upper limits for age, we resampled the participants and calculated the AUC in each resampled dataset. As shown in **Figures 2A, B**, the AUC decreased with the advancement of the upper limit age for patient resampling. For example, in the SIMPLE, CEA had an AUC of 1.00 in participants <55 years old, while its AUC decreased to 0.80 in participants <70 years old (**Figure 2A**).

## Discussion

Although several studies had investigated the effects of age and gender on serum CEA (28, 38), no study has investigated their effects on the diagnostic accuracy of pleural fluid CEA. With two independent cohorts and two statistical methods, our analysis revealed that the diagnostic accuracy of CEA decreased with increasing age. Sex had no impact on the diagnostic accuracy of CEA. Therefore, we concluded that the influence of age should be considered when interpreting the diagnostic accuracy of CEA for MPE.

We used two statistical methods to investigate the impact of age and gender on the diagnostic accuracy of CEA: subgroup analyses and resampling of participants with different upper limits. Although subgroup analysis has been frequently used to investigate the effects of age on the diagnostic accuracy of a biomarker or a model (39, 40), it has certain limitations. The primary limitation of subgroup analysis is threshold dependence, which means that its results are greatly affected by the threshold used to define different groups. In our previous work, we proposed an alternative statistical method: resampling participants with different upper limits for inclusion criteria (e.g., age). Unlike subgroup analysis, this method considers all possible thresholds to define subgroups and thus can yield more reliable results. In the subgroup analysis, the AUC of CEA in the SIMPLE revealed was significantly higher in younger patients compared with old participants. Although a higher AUC was also associated with young participants in the BUFF study, a statistically significant difference (*p*=0.377) was not demonstrated. The failure to achieve statistical significance may be attributed to the small sample size of patients with MPE in the BUFF. However, with the method of resampling participants with different age limits for enrollment, a declining trend was observed, supporting the finding in the subgroup analysis. Therefore, we concluded that age significantly affects the diagnostic accuracy of CEA, and its diagnostic accuracy for MPE should be cautiously interpreted in old patients. The findings derived from the SIMPLE cohort indicate that pleural fluid CEA demonstrates excellent diagnostic accuracy in participants aged under 55 years, and this accuracy remains strong in those under 60 years old. Therefore, the optimal age cutoff where pleural fluid CEA may be used to assist in MPE diagnosis is between 55 and 60 years.

Although previous studies have shown significantly higher CEA in men than women (28), we did not observe a significant effect of sex on the diagnostic accuracy of CEA in both cohorts. There are two plausible explanations. First, selection bias should be considered, as the participants in the previous study were healthy individuals, whereas the participants in our study are patients with pleural effusion with undetermined causes. Indeed, no studies have shown whether gender has an effect on CEA levels in patients with pleural effusion, which makes our study a valuable contribution. Second, confounding factors may influence the diagnostic accuracy of CEA in different sex, such as underlying diseases, smoking, and alcohol consumption. Indeed, some studies indicated that smokers had higher serum CEA levels than non-smokers (41, 42).

Several limitations exist in our study. The first limitation is the small sample size. The second limitation is the retrospective design of the BUFF study, which may negatively affect the completeness and accuracy of the data. Additionally, some patients may have been excluded from the study due to data incompleteness, leading to a cohort that lacks representativeness. These limitations make the BUFF study susceptible to bias.

Our study shows that the diagnostic accuracy of pleural fluid CEA declines with age, while sex has no effect. Our findings imply that age should be considered when interpreting the diagnostic accuracy of pleural fluid CEA for MPE. Given the small sample size and underrepresentation of participants in our study, it remains necessary to validate our findings in future studies.

## Data Availability

Due to ethical restrictions, we do not facilitate data sharing.
