# Supplementary material for: Influence of age and sex on the diagnostic accuracy of pleural fluid carcinoembryonic antigen for malignant pleural effusion: a post hoc analysis
Source: Front Oncol. 2025 May 6;15:1549621. doi: 10.3389/fonc.2025.1549621 (PMC12089140; doi:10.3389/fonc.2025.1549621)
Supplement: Supplementary file 1 [file Table1.docx]

**Supplementary Table 1.** Basic characteristics of patients grouped by age

| Variables | SIMPLE cohort | | |  | BUFF cohort | | |
| --- | --- | --- | --- | --- | --- | --- | --- |
|  | ≤55 years | >55 years | p |  | ≤55 years | >55 years | p |
| Gender, male/female | 15/11 | 120/64 | 0.45 |  | 32/21 | 124/58 | 0.29 |
| PF WBC, 10^6^/mL | 1919 (812–3087) | 860 (472–1780) | 0.04 |  | 1751 (794–3532) | 935 (423–2096) | 0.10 |
| PF LDH, U/L | 398 (211–686) | 231 (147–427) | 0.03 |  | 496 (258–811) | 211 (115–551) | <0.01 |
| PF glucose, mmol/L | 5.3 (4.5–6.0) | 6.0 (4.9–7.0) | 0.03 |  | 4.4 (3.7–5.5) | 5.9 (4.4–6.9) | <0.01 |
| PF ADA, U/L | 16 (7–32) | 10 (6–19) | 0.19 |  | 22 (10–39) | 12 (6–31) | 0.20 |
| PF protein, g/L | 45 (41–49) | 36 (24–43) | <0.01 |  | 35 (24–45) | 23 (16–36) | <0.01 |
| PF CEA, ng/mL | 2 (1–6) | 3 (1–36) | 0.13 |  | 1 (1–2) | 2 (1–4) | 0.24 |
| MPE/BPE | 6/20 | 85/99 |  |  | 14/39 | 36/146 |  |

Data are presented as the median (25th–75th centile) or absolute number (percentage). PF, pleural fluid; WBC, white blood cell; LDH, lactate dehydrogenase; ADA, adenosine deaminase; CEA, carcinoembryonic antigen.
